# Health insurance coverage among women of reproductive age in rural Ghana: policy and equity implications

**DOI:** 10.1186/s12961-023-01019-0

**Published:** 2023-07-14

**Authors:** Martin Ayanore, Agani Afaya, Maxwell Tii Kumbeni, Timothy Tienbia Laari, Bright Opoku Ahinkorah, Solomon Mohammed Salia, Victoria Bam, Vida Nyagre Yakong, Richard Adongo Afaya, Robert Kaba Alhassan, Abdul-Aziz Seidu

**Affiliations:** 1 Department of Health Policy Planning and Management, Fred N. Binka School of Public Health, University of Health and Allied Sciences, Ho, Ghana; 2 Mo Im Kim Nursing Research Institute, College of Nursing, Yonsei University, 50‑1, Yonsei‑ro, Seodaemun‑gu, Seoul, 03722 South Korea; 3 Department of Nursing, School of Nursing and Midwifery, University of Health and Allied Sciences, Ho, Ghana; 4 Department of Health Management and Policy, College of Public Health and Human Sciences, Oregon State University, Corvallis, USA; 5 Presbyterian Primary Health Care (PPHC), Bolgatanga, Ghana; 6 School of Public Health, Faculty of Health, University of Technology Sydney, Sydney, Australia; 7 Graduate School of Medical Sciences, Research Institute SHARE, University of Groningen, Groningen, The Netherlands; 8 Department of Nursing, Faculty of Allied Health Sciences, College of Health Sciences, Kwame Nkrumah University of Science and Technology, Kumasi, Ghana; 9 Department of Preventive Health Nursing, School of Nursing and Midwifery, University for Development Studies, Tamale, Ghana; 10 Department of Midwifery and Women’s Health, School of Nursing and Midwifery, University for Development Studies, Tamale, Ghana; 11 Centre for Health Policy and Implementation Research, Institute of Health Research, University of Health and Allied Sciences, Ho, Ghana; 12 College of Public Health, Medical and Veterinary Sciences, James Cook University, Townsville, Australia

**Keywords:** National health insurance scheme, Ghana, Women, MICS

## Abstract

**Background:**

Globally, health insurance has been identified as a key component of healthcare financing. The implementation of health insurance policies in low and middle-income countries has led to a significant increase in access to healthcare services in these countries. This study assessed health insurance coverage and its associated factors among women of reproductive age living in rural Ghana.

**Methods:**

This study used a nationally representative data from the 2017/2018 Ghana Multiple Indicator Cluster Survey (GMICS) and included 7340 rural women aged 15–49 years. Bivariate and multivariable logistic regression models were developed to assess the association between the explanatory and the outcome variable. Statistical significance was considered at *p* = 0.05.

**Results:**

The overall prevalence of health insurance coverage among rural women in Ghana was 51.9%. Women with secondary (aOR = 1.72, 95% CI: 1.38–2.14) and higher education (aOR = 4.57, 95% CI: 2.66–7.84) were more likely to have health insurance coverage than those who had no formal education. Women who frequently listened to radio (aOR = 1.146, 95% CI: 1.01–1.30) were more likely to have health insurance coverage than those who did not. Women who had a child (aOR = 1.81, 95% CI: 1.50–2.17), two children (aOR = 1.59, 95% CI: 1.27–1.98), three children (aOR = 1.41, 95% CI: 1.10–1.80), and five children (aOR = 1.36, 95% CI: 1.03–1.79) were more likely to have health insurance coverage than those who had not given birth. Women who were pregnant (aOR = 3.52, 95% CI: 2.83–4.38) at the time of the survey, and women within the richest households (aOR = 3.89, 95% CI: 2.97–5.10) were more likely to have health insurance coverage compared to their other counterparts. Women in the Volta region (aOR = 1.36, 95% CI: 1.02–1.81), Brong Ahafo region (aOR = 2.82, 95% CI: 2.20–3.60), Northern region (aOR = 1.32, 95% CI: 1.02–1.70), Upper East region (aOR = 2.13, 95% CI: 1.63–2.80) and Upper West region (aOR = 1.56, 95% CI: 1.20–2.03) were more likely to have health insurance coverage than those in the Western region.

**Conclusion:**

Although more than half of women were covered by health insurance, a significant percentage of them were uninsured, highlighting the need for prompt policy actions to improve coverage levels for insurance. It was found that educational level, listening to radio, parity, pregnancy status, wealth quintile, and region of residence were factors associated with health insurance coverage. We recommend better targeting and prioritization of vulnerability in rural areas and initiate policies that improve literacy and community participation for insurance programs. Further studies to establish health policy measures and context specific barriers using experimental designs for health insurance enrolments are required.

## Introduction

Globally, enrolment for social health insurance is one of the financing mechanisms Governments use to raise revenues to fund healthcare [1]. The implementation of health insurance policies in low- and middle-income countries (LMICs) has led to a significant increase in access to healthcare services in these countries [2]. Globally, there are different models for health financing, suggesting a not one-size-fit all approach to risk pooling. Across LMICs, a mix of different health financing approaches are known and apply, from central Government allocations (taxation), social or voluntary health insurance, and others [3].

In Ghana, the government introduced the National health insurance scheme (NHIS) in 2003 as a pro-poor policy to guarantee financial risk protection and remove financial barriers as a limitation for accessing and utilizing healthcare services [2]. Under the NHIS, premium payments, formal sector worker’s contributions through social security contributions, a 2.5% health Insurance Levy, and external donor funding are other modes of funding [4]. Membership renewals is on an annual basis. The scheme provides categories of persons exempted from paid membership; pregnant women, older adults aged 70 years and above, indigents, patients with mental problems, and other classifications determined by the Minister for Gender [4].

Following the successful rollout of the NHIS and to improve efforts to scale-up maternal and child health outcomes during the Millennium Development Goals (MDGs) era, the Government of Ghana further introduced the free maternal healthcare policy [5]. The free maternal healthcare policy was introduced in 2008 [6]. Under the free maternal policy, women of reproductive age are entitled to free services throughout pregnancy, childbirth, and postpartum care, irrespective of ones geographical location in Ghana. Specifically, the policy provides the opportunity for all pregnant women to have free registration under the NHIS and to benefit from free maternal care throughout the pregnancy and 3 months postpartum [7]. Despite the implementation of the free maternal health care policy, most women of reproductive age do not still enroll in the NHIS or other private schemes due to a myriad of factors [6–8].

The Ghanaian population consists of approximately 49% of people living in rural areas with limited socio-economic opportunities [9] and there are significant inequities between urban and rural areas in terms of the availability of health facilities and potable water [9]. The overall poverty rate per capita in urban areas is 10% while in rural areas it is as high as 39% [9]. These rural–urban inequity has been observed to also influence the enrolment of women in rural areas into the NHIS, as studies in Ghana have shown that socio-economic status is a major determinant of NHIS enrolment [10, 11] and maternal health care utilization [12]. Though the design and implementation of the NHIS was envisioned as a pro-poor policy, concerns that the policy largely benefits the rich instead of the poor have been reported [13].

Evidence has shown that rural residents are less likely to have health insurance coverage, relative to those in urban areas [13–16]. In Ghana, data from the Ghana Demographic Health Surveys showed that approximately 66% and 53% of women and men respectively of all ages were covered by health insurance in Ghana based on data in 2014 [17]. In the year 2020, the Government of Ghana reinvigorated efforts towards the goal of achieving 80% of essential services through ambitious policy reforms, including maternal and child health [18]. These strategies also included accelerating progress towards Universal Health Coverage (UHC), with strategies of improving the national health insurance scheme to reduce financial barriers to health care, particularly among vulnerable and low socioeconomic groups [19].

In this regard, one of the key target populations for health insurance coverage in Ghana is women of reproductive age, particularly those living in rural areas. This is in view of their vulnerability to poor maternal and reproductive health outcomes [20]. Despite previous studies on the determinants of NHIS enrolment among women and general population in Ghana using DHS [9, 17, 21, 22], there is still need for building an extensive body of knowledge on equity of access for rural reproductive age women's health insurance coverage and its determinants. Limited studies have examined NHIS coverage using recent evidence from the Ghana Multiple Indicator Cluster Survey (GMICS) with the aim of identifying major determinants that predict insurance coverage among rural women in Ghana.

To fill this knowledge gap in Ghana, the current study sought to use nationally representative data to assess the prevalence and factors associated with health insurance coverage among women of reproductive age in rural Ghana. This specific group is relevant since reproductive age women insurance status can influence family level insurance status, children and other older adults. Women with health insurance coverage are likely to use more health care services along their life course, thus we considered understanding this specific group. The results from this study would provide additional insights to context specific factors that shape rural women access to insurance and healthcare and its implications for meeting health equity goals in Ghana across similar context in LMICs.

## Methods

### Data source

This study used a nationally representative data from the 2017/2018 Ghana Multiple Indicator Cluster Survey (MICS). The survey was conducted by the Ghana Statistical Service in collaboration with the Ministry of Health, Ghana Health Service, the Ministry of Education, and the Ministry of Gender, Children and Social Protection. MICS received funding and technical support from the United Nations International Children’s Fund (UNICEF), the Korea International Cooperation Agency (KOICA), the United Nations Development Program (UNDP), and other international agencies [23]. MICS measures critical indicators that provide countries with data to guide the implementation of programs, policies, and national development plans and other international commitments. The survey employed a multistage stratified sampling design to select households from both rural and urban communities of the previous ten regions of Ghana. The first stage involved identifying and selecting enumeration areas with probability proportional to size based on the 2010 Ghana Population and Housing Census. The primary sampling unit for the survey was the enumeration areas. The second stage involved the selection of households from the enumeration areas, and this was done by using systematic random sampling. From each selected household, data on women of reproductive age were recruited for the survey. This study selected women who had complete cases on all the variables of interest and that gave us 7340 reproductive age women (15–49 years) in rural Ghana.

### Measures

#### Outcome variable

The outcome variable of this study was health insurance coverage among women of reproductive age in rural Ghana. The question was asked “Are you covered by any health insurance?” and the responses were categorized as “No” and “Yes”. Women who were covered by health insurance were defined as those who were registered with any health insurance organization and their health insurance was valid at the time of the MICS survey.

#### Explanatory variables

The explanatory variables included the age of the women, marital status, ethnicity, educational status, parity, pregnancy status, frequency of reading newspaper or magazine, frequency of listening to the radio, frequency of watching television, wealth quintile**,** and region of residence. The selection of the explanatory variables was based on relevant related literature [15–17, 24] and the availability of the variables in the dataset.

The age of women was coded in age groups as 15–19, 20–24, 25–29, 30–34, 35–39, 40–44, and 45–49; marital status was categorized as never married, cohabitating, and married; ethnicity was coded as Akan, Ga/Dangme, Ewe, Guan, Gruma, Grusi, Mande, Mole-Dagbani, and others; educational status was coded as no education, primary, middle, secondary, and higher education; Parity was coded as 0, 1, 2, 3, and ≥ 4; Pregnancy status was coded as not pregnant and pregnant; frequency of reading newspaper or magazine, frequency of listening to the radio, and frequency of watching television were categorised as “not at all”, “less than once a week”, “at least once a week”, and “almost every day”; wealth quintile was coded as poorest, poorer, middle, richer and richest, and region was categorized as Western, Central, Greater Accra, Volta, Eastern, Ashanti, Brong Ahafo, Northern, Upper East, and Upper West.

### Statistical analyses

The statistical analysis of this study was conducted using Stata version 16.0 (StataCorp, College Station, TX, USA). Descriptive statistics of the study population such as frequencies and percentages were used to present the explanatory variables. Bivariate logistic regression was conducted to determine the association between each  explanatory variable and the outcome variable (health insurance coverage). We then built a multivariable logistic regression model to assess the association between all the explanatory variables and the outcome variable. Multicollinearity was checked using the Variance Inflation Factor (VIF) and all the explanatory variables had VIF scores less than 10.0. The study sample was weighted, and the surveyset svy command in Stata was used in the analysis to account for the survey’s complex nature and the generalizability of the findings.

### Ethical consideration

This study did not require ethical approval because we used publicly available data from MICS. Details of ethics approval for MICS is available at https://mics.unicef.org/tools. Written or verbal consent was sought before data collection from participants. This survey followed the standard guidelines for conducting human research as stated in the declaration of Helsinki.

## Results

### Sociodemographic characteristics of respondents

A total of 7340 rural women were sampled for this study. The overall health insurance coverage among rural women was 51.9% while a substantial number (48.1%) were non-insured. The largest age group of the rural women (21.4%) were between the ages of 15–19 years and most of the women (61.3%) were married. Among the rural women, a few of them (1.9%) had higher education while about 40.9% had middle school education. In terms of parity, the largest group (39.8%) had four or more children while a few of them (7.4%) were pregnant at the time of the study. The majority (30.6%) of the women were within the poorest wealth quintile and the least (8.4%) were within the richest household (Table 1).

**Table 1:** Sociodemographic characteristics of respondents

Variables	Weighted N (7340)	Weighted %	NHIS coverage	
			No (%) 48.1	Yes (%) 51.9
Age (in years)				
15–19	1569	21.4	52.8	47.2
20–24	1105	15.1	43.2	56.8
25–29	1088	14.8	42.2	57.8
30–34	1015	13.8	50.1	49.9
35–39	1046	14.2	48.5	51.5
40–44	851	11.6	47.2	52.8
45–49	665	9.1	52.5	47.5
Marital status				
Never in union	2180	29.7	50.4	49.6
Cohabitation	659	9.0	51.2	48.8
Married	4501	61.3	46.6	53.4
Educational status				
No education	1875	25.6	50.0	49.0
Primary	1569	21.4	53.1	46.9
Middle	3003	40.9	48.4	51.6
Secondary	751	10.2	35.9	64.1
Higher	142	1.9	15.5	84.5
Ethnicity				
Akan	3188	43.4	47.4	52.6
Ga/Dangme	559	7.6	57.1	42.9
Ewe	826	11.3	54.5	45.5
Guan	412	5.6	32.1	67.9
Gruma	382	5.2	56.6	43.4
Mole Dagbani	1271	17.3	44.7	55.3
Grusi	204	2.8	46.1	53.9
Mande	23	0.3	55.6	44.4
Others	472	6.4	44.9	55.1
Parity				
0	1911	26.0	51.4	48.6
1	909	12.4	35.8	64.2
2	795	10.8	42.8	57.2
3	803	11.0	49.1	50.9
4	797	10.9	49.2	50.8
5	691	9.4	49.9	50.1
6	623	8.5	50.4	49.6
7	373	5.1	57.0	43.0
8	224	3.0	55.9	44.1
9+	213	2.9	61.1	38.9
Pregnancy status				
Not pregnant	6798	92.6	50.4	49.6
Pregnant	542	7.4	19.5	80.5
Frequency of reading newspaper or magazine				
Not at all	6969	94.9	48.6	51.4
Less than once a week	232	3.2	43.8	56.2
At least once a week	113	1.5	36.2	63.8
Almost every day	26	0.4	28.8	71.2
Frequency of listening to the radio				
Not at all	2734	37.3	48.8	51.2
Less than once a week	1313	17.9	51.2	48.8
At least once a week	1403	19.1	46.1	53.9
Almost every day	1890	25.7	46.6	53.4
Frequency of watching television				
Not at all	2796	38.1	51.4	48.6
Less than once a week	954	13.0	49.6	50.4
At least once a week	1140	15.5	46.9	53.1
Almost every day	2450	33.4	44.5	55.5
Wealth quintile				
Poorest	2248	30.6	54.5	45.5
Poor	1,994	27.2	51.5	48.5
Middle	1546	21.1	45.2	54.8
Richer	932	12.7	41.9	58.1
Richest	620	8.4	31.0	69.0
Region				
Western	893	12.2	48.5	51.5
Central	778	10.6	58.5	41.5
Greater Accra	234	3.2	60.8	39.2
Volta	814	11.1	44.4	55.6
Eastern	938	12.8	55.1	44.9
Ashanti	1457	19.8	45.4	54.6
Brong Ahafo	666	9.1	30.0	70.0
Northern	962	13.1	52.3	47.7
Upper East	338	4.6	39.1	60.9
Upper West	260	3.5	49.2	50.8

Data Source: 2017/2018 Ghana Multiple Indicator Cluster Survey

### Factors associated with health insurance coverage among women of reproductive age in rural Ghana

Table 2 depicts the factors associated with health insurance coverage among women in rural Ghana. From the multivariable logistic regression analysis, we observed that women with primary (aOR = 1.26, 95% CI: 1.04-1.41), secondary (aOR = 1.81, 95% CI: 1.45–2.25) and higher education (aOR = 4.77, 95% CI: 2.78–8.19) were more likely to have health insurance coverage than those who had no formal education. Women who frequently listened to radio (aOR = 1.146, 95% CI: 1.01–-1.30) were more likely to have health insurance coverage than those who did not listen to radio. Women who had a child (aOR = 1.81, 95% CI: 1.50–2.17), two children (aOR = 1.59, 95% CI: 1.27–1.98), three children (aOR = 1.41, 95% CI: 1.10–1.80) and five children (aOR = 1.36, 95% CI: 1.03–1.79) were more likely to have health insurance coverage than those who had not given birth. Women who were pregnant (aOR = 3.54, 95% CI: 2.84–4.42) at the time of the survey were more likely to have health insurance coverage than those who were not pregnant. We noticed that as the household wealth increased the more likely the women would have health insurance coverage. Specifically, women within the richest household (aOR = 3.84, 95% CI: 2.93–5.03) were over three times more likely to have health insurance coverage than those in the poorest households. Women in the Volta region (aOR = 1.36, 95% CI: 1.02–1.81), Brong Ahafo region (aOR = 2.82, 95% CI: 2.20–3.60), Northern region (aOR = 1.32, 95% CI: 1.02–1.70), Upper East region (aOR = 2.13, 95% CI: 1.63–2.80) and Upper West region (aOR = 1.56, 95% CI: 1.20–2.03) were more likely to have health insurance coverage than those in the Western region.

**Table 2:** Bivariate and multivariable analysis of the factors associated with health insurance coverage among women of reproductive age in rural Ghana

Variables	cOR [95% CI]	aOR [95% CI]
Age (in years)		
15–19	Ref	Ref
20–24	1.34*** [1.16–1.55]	0.92 [0.76–1.09]
25–29	1.36*** [1.16–1.59]	0.90 [0.72–1.13]
30–34	1.08 [0.92–1.28]	0.83 [0.65–1.08]
35–39	1.04 [0.88–1.22]	0.95 [0.72–1.24]
40–44	1.05 [0.88–1.24]	0.98 [0.74–1.29]
45–49	0.85 [0.71–1.02]	0.88 [0.66–1.18]
Educational status		
No education	Ref	Ref
Primary	0.90** [0.78–1.02]	0.95 [0.79–1.07]
JSS/JHS/Middle	1.18*** [1.06–1.32]	1.26** [1.04–1.41]
SSS/SHS/ Secondary	1.81*** [1.53–2.14]	1.81*** [1.45–2.25]
Higher	6.40*** [3.96–10.34]	4.77*** [2.78–8.19]
Ethnicity		
Akan	Ref	Ref
Ga/Dangme	0.67*** [0.54–0.84]	0.82 [0.64,1.06]
Ewe	0.75** [0.63–0.88]	0.73** [0.57,0.92]
Guan	1.89*** [1.48–2.42]	1.81*** [1.34,2.43]
Gruma	0.68*** [0.56[0.85]	0.88 [0.67,1.16]
Mole Dagbani	1.11 [0.98–1.25]	1.14[0.92,1.41]
Grusi	1.04 [0.85–1.29]	0.99 [0.76,1.30]
Mande	0.72 [0.91–1.54]	0.76 [0.34,1.70]
Others	1.10 [0.91–1.32]	1.19 [0.93,1.52]
Parity		
0	Ref	Ref
1	1.59*** [1.37–1.86]	1.81*** [1.50,2.17]
2	1.23* [1.04–1.45]	1.59*** [1.27,1.98]
3	1.10 [0.93–1.30]	1.41** [1.10,1.80]
4	0.97 [82–1.15]	1.27 [0.97,1.66]
5	0.95 [0.79–1.13]	1.36* [1.03,1.79]
6	0.93 [0.77–1.11]	1.29 [0.96,1.74]
7	0.71** [0.57–0.89]	1.05 [0.76,1.44]
8	0.74* [0.56–0.98]	1.13 [0.77,1.64]
9+	0.60** [0.45–0.81]	0.97 [0.66,1.43]
Pregnancy status		
Not pregnant	Ref	Ref
Pregnant	3.43*** [2.78–4.22]	3.54*** [2.84–4.42]
Frequency of reading newspaper or magazine		
Not at all	Ref	Ref
Less than once a week	1.26 [0.96–1.66]	0.85 [0.63–1.14]
At least once a week	1.80** [1.23–2.61]	1.21 [0.79–1.86]
Almost every day	1.75 [0.81–3.77]	1.06 [0.47–2.41]
Frequency of listening to the radio		
Not at all	Ref	Ref
Less than once a week	0.89 [0.78–1.02]	0.94 [0.81–1.09]
At least once a week	1.05 [0.92–1.19]	0.99 [0.86- 1.15]
Almost every day	1.19** [1.06–1.34]	1.15* [1.01–1.30]
Frequency of watching television		
Not at all	Ref	Ref
Less than once a week	0.9 [0.79–1.07]	0.88 [0.74–1.05]
At least once a week	1.21** [1.05–1.39]	1.07 [0.91–1.26]
Almost every day	1.25*** [1.12–1.40]	0.88 [0.76–1.02]
Wealth quintile		
Poorest	Ref	Ref
Poorer	1.08 [0.96–1.22]	1.30*** [1.14–1.50]
Middle	1.41*** [1.24–1.61]	1.87*** [1.59–2.21]
Richer	1.58*** [1.34–1.87]	2.14*** [1.74–2.63]
Richest	2.99*** [2.39–3.74]	3.84*** [2.93–5.03]
Region		
Western	Ref	Ref
Central	0.76* [0.62–0.95]	0.64*** [0.51–0.80]
Greater Accra	0.75 [0.53–1.0]	0.69 [0.46–1.04]
Volta	1.22 [0.99–1.50]	1.36* [1.02–1.81]
Eastern	0.82 [0.67–1.02]	0.91 [0.72–1.15]
Ashanti	1.25* [1.01–1.54]	1.18 [0.72–1.15]
Brong Ahafo	2.53*** [2.02–3.18]	2.82*** [2.20,3.60]
Northern	0.97 [0.80–1.18]	1.32* [1.02–1.70]
Upper East	1.62*** [1.32–1.98]	2.13*** [1.63–2.80]
Upper west	1.16 [0.96–1.40]	1.56*** [1.20–2.03]

Exponentiated coefficients; 95% confidence intervals in brackets; cOR: crude odds ratios; aOR: adjusted odds ratios; CI: Confidence Interval; *p < 0.05, **p < 0.01, ***p < 0.001; 1 = Reference category

## Discussion

Using data from the 2017/2018 GMICS, we examined health insurance coverage and factors associated with coverage among women of reproductive age in rural Ghana. Slightly more than half of rural women were covered for health insurance. Our study revealed that the odds of health insurance coverage were higher among educated women (secondary and higher education), women who frequently listened to radio, those with parity levels of 1,2,3, and 5 children, pregnant women, and women within the richest household. Women in the Volta, Brong Ahafo, and Northern, Upper East, and Upper West regions were more likely to have health insurance coverage than those in the Western region.

Our findings revealed that the overall coverage for health insurance among rural women was 51.9%. Albeit this prevalence is relatively higher than those reported in some countries in SSA [15, 16, 24], the prevalence in this study is however lower compared to Ghana’s NHIS objective of ensuring universal health insurance coverage among persons resident in the country by 2030 [25]. Only 54.4% of the total population is covered by the NHIS, with informal sector contributing less than 40 [18]. To accelerate efforts to achieve the Government of Ghana’s target of 80% coverage for essential health services and by extension meet targets for universal health coverage (UHC) by 2030, a high population coverage for health insurance is required. Besides, efforts to scale-up primary health care services, particularly among rural population groups will be hampered if coverage for rural insurance is not improved. Policy options to minimize financial barriers to health, particularly among vulnerable population groups is increasingly required. Furthermore, rural women from this study were more likely to be covered with health insurance as their educational level increased. Thus, those with secondary or higher education had higher odds of health insurance coverage than those who had no formal education. This is consistent with studies in Namibia [26], Indonesia [27], and Malawi [28], and is further supported by a recent multi-national study in East Africa [29]. This positive correlation might have occurred because compared with women with no education, women with higher education generally have increased access to health information including information on health insurance. Also, women with higher education are more likely to be employed with high income and hence, able to afford health insurance premiums. Again, women with higher education are more likely to be employed in the formal sector with contributions to the Social Security and National Insurance Trust (SSNIT). In Ghana, SSNIT contributors are automatically covered by the NHIS and exempt from paying premiums [25].

In line with previous studies in Kenya [30] and Zambia [31], our results revealed a significant association between the frequency of listening to radio and health insurance coverage. The odds of health insurance coverage was higher among women who frequently listened to radio than those who did not. This could be attributed to the fact that women who frequently listen to the radio are exposed to information on a wide range of health interventions such as health insurance. Thus, rural women who listen to radio, especially programs that focus on health and well-being may hear some relevant information such as the benefits of being covered by health insurance, and how to obtain coverage. This increased awareness could encourage women to subscribe to health insurance. This finding points to the critical role that the radio plays in increasing public awareness and promoting the uptake of health interventions such as health insurance especially among rural folks.

The current study revealed that parity was significantly associated with health insurance coverage. Women who had one, two, and three or more children were more likely to have health insurance coverage than those who had not given birth. This finding corroborates earlier studies in Ghana [32, 33] that identified an association between parity and health insurance coverage. One plausible explanation for this correlation could be that nulliparous women are least likely to seek health care because they do not require maternal health care services and hence, are less likely to subscribe to a health insurance scheme [17]. On the contrary, since women who have ever given birth were more likely to require maternal health care services which are provided free of charge to NHIS subscribers, they were more inclined to subscribe to health insurance.

We also found that women who were pregnant at the time of the survey were more likely to have health insurance coverage than those who were not pregnant. Amu et al. [34] posited that many sub–Sharan African countries with high health insurance coverage have prioritized women in their health insurance policies. The positive link between pregnancy and health insurance coverage in Ghana can be attributed to the national policy on free maternal care, which provide opportunity for comprehensive range of care services from conception till postpartum once women are insured under the NHIS [35, 36].

Consequently, pregnant women often take advantage of this policy to enroll in the NHIS to avoid out-of-pocket payments for perinatal care services [37].

Health insurance coverage from this study increased with increasing household wealth, corroborating the evidence that individual or household wealth is a predictor for NHIS uptake. Women within the richest household were more likely to have health insurance coverage than those in the poorest households. This finding corroborates previous studies in Namibia [38] and Peru [39] and a recent multi-national study in SSA [26].

Albeit Ghana’s NHIS premium exemptions includes persons aged 70 years and above and those classified as “indigents” (the core poor) [25]. This means that the vulnerable and low socio-economic groups suffer financially to enroll or renew their membership to the NHIS. In Ghana, premium payments remain a hindrance for NHIS enrolments in Ghana. Kwarteng et al. [13] posited that the cost of the premium is still a major obstacle to NHIS registration in Ghana. For women within the lowest wealth quartiles, yet have no or minimal disposable income, high premium payments may be a deterrent and will make it extremely difficult for women in the poorest households to subscribe to health insurance.

Our study also identified a geographical pattern regarding health insurance coverage among rural women in Ghana. Women in the Volta, Brong Ahafo, Northern, Upper East, and Upper West regions were more likely to have health insurance coverage than those in the Western region (coastal zone). Regional variations have also been reported in Kenya [30], and Nigeria [24]. Previous studies in Ghana established that health insurance coverage was higher among women in the middle and northern zones while the coastal zone recorded the least coverage [33, 40]. This is further corroborated by the NHIS report that the Western region recorded 9.5% of NHIS enrolment [41] and also had the least coverage among the stated regions [42]. While this study did not investigate reasons associated with differences in NHIS coverage across regions, a previous study by Ayanore et al. (2019) on reasons for not being insured under the NHIS mentioned mistrust as a key factor that determines enrolments rates in Ghana [17]. While several reasons can also account for this, other studies in Ghana also report that differences in socioeconomic and demographic factors are responsible for variations in NHIS enrolment rates across the regions of Ghana [9, 43].

Both parity (1–3 births) and pregnancy status predicted coverage for insurance among rural women and indicating an increased demand for healthcare services among reproductive-age women along their life course. With the existence of the free maternal health policy, the proportion of women insured require further investigation. Despite this increased demand along their life course, the coverage of approximately 52% for insurance means a sizable number of women may be losing out in insurance use to meet essential maternal and reproductive care needs, particularly along the continuum of care for both mothers and children. Further studies that apply experimental designs are required to establish context specific reasons and equity barriers to improve health insurance coverage.

Lastly, wealth status has long been established as a strong predictor not only for insurance but overall health and well-being [44, 45]. Vulnerable and poor rural households in this study were less likely to be covered for insurance. In Ghana, studies are required to establish the nexus of health and poverty, or vulnerability based on rural and urban status and how these factors impact health insurance coverage. An understanding of the complex relationship between health and poverty is crucial for policy interventions and for defining the benefit package under the NHIS to address the need of vulnerable and poor rural households.

### Strengths and Limitations

The use of a nationally representative dataset makes our study generalizable to rural context in Ghana. Nonetheless, the study used cross-sectional data and causal interpretations cannot be inferred. In addition, the study was limited to only variables in the dataset, hence key factors such as perception of quality of healthcare for health insurance subscribers could not be assessed.

## Conclusion

More than half of rural women in this study were covered by health insurance. Predictors for coverage includes educational level, parity, currently pregnant, wealth quintile, and geography (region). To accelerate progress towards the attainment of UHC goals in Ghana, there is the need to address both vertical and horizontal equity needs that influence coverage for insurance. We recommend better targeting and prioritization of vulnerability in rural areas and initiate policies that improve literacy and community participation for insurance programs. Further studies to establish health policy measures and context specific barriers using experimental designs for health insurance enrolments are required.

## Data Availability

The datasets used for this study is openly available and can be accessed via https://mics.unicef.org/tools.

## Abbreviations

aOR: Adjusted odds ratio
CIs: Confidence intervals
COR: Crude odds ratio
GMICS: Ghana Multiple Indicator Cluster Survey
KOICA: Korea International Cooperation Agency
LMICs: Low and middle-income countries
NHIA: National Health Insurance Authority
NHIS: National Health Insurance Scheme
SDGs: Sustainable Development Goals
SSNIT: Social Security and National Insurance Trust
UNDP: United Nations Development Program
UNICEF: United Nations International Children’s Fund
VIF: Variance Inflation Factor
